# Applications of various range shifters for proton pencil beam scanning radiotherapy

**DOI:** 10.1186/s13014-021-01873-8

**Published:** 2021-08-06

**Authors:** Haibo Lin, Chengyu Shi, Sheng Huang, Jiajian Shen, Minglei Kang, Qing Chen, Huifang Zhai, James McDonough, Zelig Tochner, Curtiland Deville, Charles B. Simone, Stefan Both

**Affiliations:** 1 New York Proton Center, New York, 10035 USA; 2 Mayo Clinic, Phoenix, AZ 85054 USA; 3 Department of Radiation Oncology, University of Pennsylvania, Philadelphia, PA 19104 USA; 4 Department of Radiation Oncology and Molecular Radiation Sciences, Johns Hopkins University, Baltimore, MD 21231 USA; 5 Department of Radiation Oncology, University Medical Center Groningen, Groningen, 9713 GZ Netherlands

**Keywords:** Proton therapy, Pencil beam scanning (PBS), Range shifter, Bolus

## Abstract

**Background:**

A range pull-back device, such as a machine-related range shifter (MRS) or a universal patient-related range shifter (UPRS), is needed in pencil beam scanning technique to treat shallow tumors.

**Methods:**

Three UPRS made by QFix (Avondale, PA, USA) allow treating targets across the body: U-shaped bolus (UB), anterior lateral bolus (ALB), and couch top bolus. Head-and-neck (HN) patients who used the UPRS were tested. The in-air spot sizes were measured and compared in this study at air gaps: 6 cm, 16 cm, and 26 cm. Measurements were performed in a solid water phantom using a single-field optimization pencil beam scanning field with the ALB placed at 0, 10, and 20 cm air gaps. The two-dimensional dose maps at the middle of the spread-out Bragg peak were measured using ion chamber array MatriXX PT (IBA-Dosimetry, Schwarzenbruck, Germany) located at isocenter and compared with the treatment planning system.

**Results:**

A UPRS can be consistently placed close to the patient and maintains a relatively small spot size resulting in improved dose distributions. However, when a UPRS is non-removable (e.g. thick couch top), the quality of volumetric imaging is degraded due to their high Z material construction, hindering the value of Image-Guided Radiation Therapy (IGRT). Limitations of using UPRS with small air gaps include reduced couch weight limit, potential collision with patient or immobilization devices, and challenges using non-coplanar fields with certain UPRS. Our experience showed the combination of a U-shaped bolus exclusively for an HN target and an MRS as the complimentary device for head-and-neck targets as well as for all other treatment sites may be ideal to preserve the dosimetric advantages of pencil beam scanning proton treatments across the body.

**Conclusion:**

We have described how to implement UPRS and MRS for various clinical indications using the PBS technique, and comprehensively reviewed the advantage and disadvantages of UPRS and MRS. We recommend the removable UB only to be employed for the brain and HN treatments while an automated MRS is used for all proton beams that require RS but not convenient or feasible to use UB.

## Background

Proton therapy has developed rapidly in recent years and the pencil beam scanning (PBS) delivery technique has been introduced to many new proton therapy centers [1]. It is well known that the PBS technique can provide more conformal dose distribution to the target volume than the traditional proton double scattering and uniform scanning techniques [2]. The potential of the proton PBS technique to generate highly conformal dose distributions relies heavily on the ability to maintain small beam spot sizes [3, 4]. Many factors can affect the spot sizes such as the proton energy, the treatment depth, and the air gap between a beam modifier and the patient [5, 6].

Due to the extraction and transport efficiency, all current PBS systems have limitations in their minimum deliverable energy. For example, the IBA system (Ion Beam Applications, Louvain-La-Neuve, Belgium) provides minimum proton energy of 60–100 MeV [3]. The traditional way to treat targets shallower than the minimum range of protons is to use machine-mounted range shifters (MRS). The range shifters (RS) are a slab of uniform material, usually made of plastic, to pull back the range of protons to the desired treatment depth. The MRS has limitations in clinical applications. Firstly, the air gap between the MRS and the isocenter plane for the non-movable nozzle is usually considerable (e.g. > 40 cm). The air gap to the isocenter plane can be reduced to about 25 cm for the movable nozzle before RS hits the couch. It is well known that the air gap distance has a strong impact on the spot size [7]. Hence, to maintain small spot sizes, it is essential to keep the air gap as small as possible. Secondly, it is very challenging for the existing proton analytical algorithms to accurately model this large range of air gaps when an MRS is used. For example, the proton convolution superposition (PCS) in Eclipse TPS (Varian Medical System, Palo Alto, CA, USA) underestimates the spot size distal to an RS because the spot geometric broadening along the air gap between the RS and the patient is not fully considered [7]. This introduces considerable inaccuracies, namely up to 30% errors on spot sizes when the position of RS differs more than 15 cm from the commissioning position [8]. Ding et al. [9] reported their commissioning results of RS for PBS with RayStation TPS (RaySearch Laboratories, Stockholm, Sweden) and recommend using air gaps from 5–10 cm to maintain accurate dose calculations.

Both et al. [3] proposed using a universal bolus that can be placed very close to the patient as an alternative to the MRS. When a patient bolus is employed with air gaps from 2 to 10 cm, the spot size remains nearly identical to the open field. This universal bolus has been successfully implemented for the treatment of brain and HN patients.

Inspired by the success of the aforementioned work by Both et al. [3], a new universal bolus (U-shaped bolus) with an increased dimension in the patient’s anterior–posterior direction, an indexed attachable design to allow flexible lateral beam angles and an improved reproducibility on the placement of the bolus was developed. Furthermore, two customized universal patient-related range shifters (UPRS) were designed and implemented to treat different disease sites beyond the brain and HN. In this work, we present the development and clinical implementations of the new U-Shaped bolus and the two new UPRS. We comprehensively reviewed the advantages and disadvantages between UPRS and MRS. Although this is a single institute experience, we hope our experience will provide useful guidance for other proton institutes on choosing the appropriate RS technique.

## Materials and methods

The Roberts Proton Therapy Center at the University of Pennsylvania currently has five PBS treatment rooms, including four gantries and one fixed beam room (IBA, Louvain-La-Neuve, Belgium). Due to low beam efficiency (< 1%), the proton PBS system runs with minimum proton energy of 100 MeV, which corresponds to a proton range of 7.71 cm water equivalent thickness (WET). Other proton PBS delivery systems also have a similar limitation, but the threshold energy varies. To overcome this limitation, different designs of range shifters are used to fulfill all clinical needs.

**Fig. 1:**
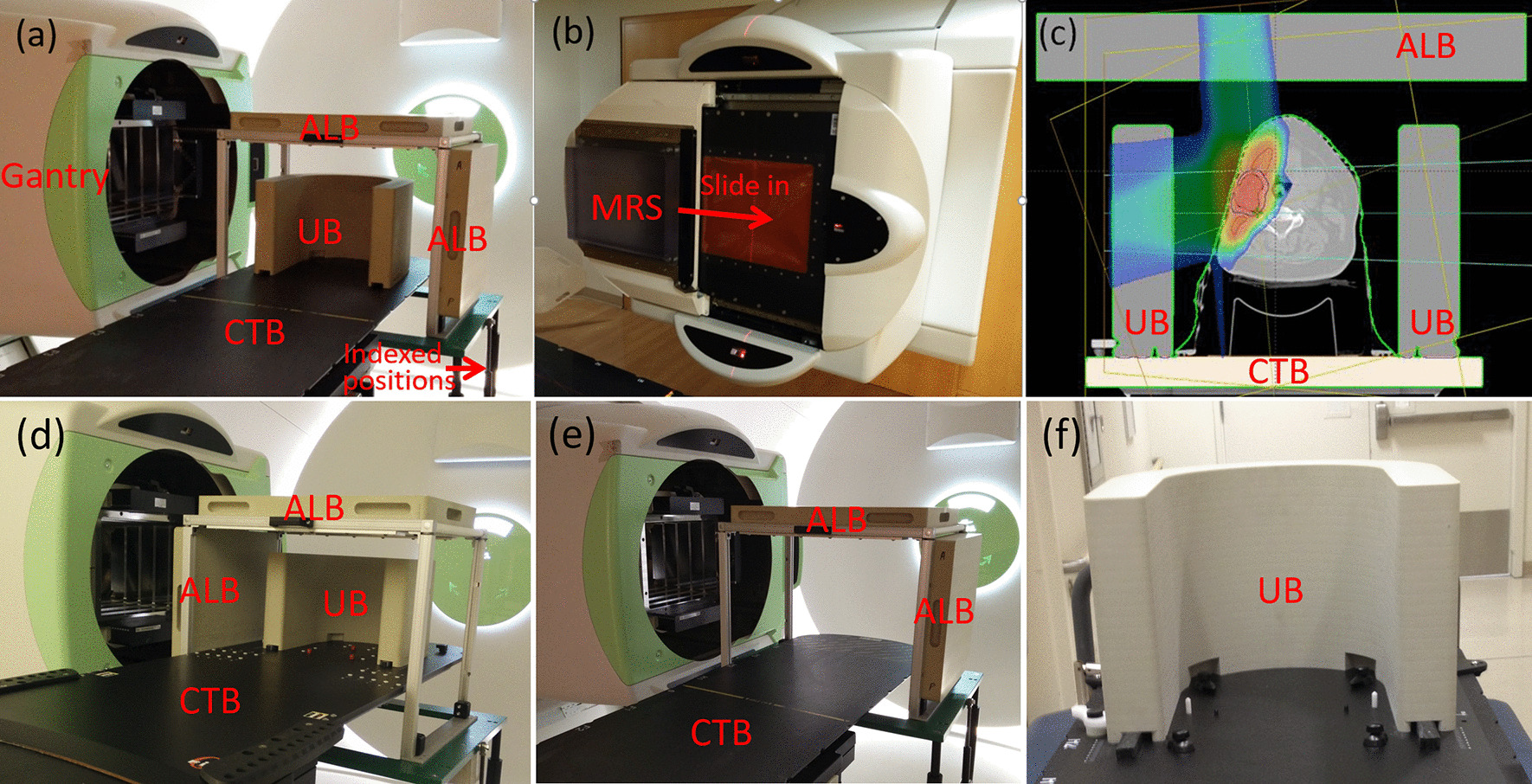
**a** Patient-related range shifters: U-shaped bolus (UB) indexed on couch top (two tracks on the couch top to let the UB slide in and index its position along the cranial-caudal direction), anterior-lateral bridge bolus (ALB) on a movable cart with indexed height (can move up and down), couch top bolus (CTB). The UB is applied in the clinic to treat brain, head, and neck targets. It allows anterior and left lateral beam in **a** and the ALB on the left side can be manually moved to the right side when it is needed (**d**). ALB is used across the patient body when anterior and lateral fields are planned. CTB is used for all treatments whenever posterior fields are used. **b** MRS mounted on the nozzle on a fixed beam nozzle. **c** An example of a head and neck patient planned with UB, ALB, and CTB in the planning images, but only UB and ALB were in the beam paths. **d** the same setup to **a** but the left ALB is moved to the right side. **e** ALB and CTB. **f** UB only

As shown in Fig. 1a, three UPRS made by QFix (Avondale, PA, USA) allow treating targets across the body: U-shaped bolus (UB), anterior lateral bolus (ALB), and couch top bolus (CTB), while the MRS (from IBA) is mounted either on movable (Fig. 1a) or fixed nozzle (Fig. 1b). Homogeneous water equivalent materials of uniform thickness with different densities were employed for UPRS. The materials and components of MRS and UPRS are summarized in Table 1. To verify the uniformity of the slab, the RSs were scanned using computed tomography (CT) scanner (Siemens Somaton Sensation, Munich, Germany) during the acceptance and commissioning. No noticeable structure heterogeneity such as holes or gaps was found by visual inspection of the 3D CT images of the RSs. Their WETs are measured by Zebra (IBA-Dosimetry, Schwarzenbruck, Germany) at multiple locations with a given RS and derived from the measured range differences of the same proton beam with and without RS present in the beam path.

**Table 1:** Materials and components of range shifter

Range shifter	Materials	Density (g/cc)	Thickness (cm)	Components
USB	Polyurethane	1.2	6.5	9.4% H, 64.2% C, 3.1% N, 23.3% O
ALB	Polyurethane	1.2	7.4	9.4% H, 64.2% C, 3.1% N, 23.3% O
CTB	G-10	1.85	3.5	54% O, 38% Si, 8% Al, and 1 mm C on top and bottom
IBA MRS	Lexan	1.2	6.48	5.5% H, 75.6% C, 18.9% O

H: hydrogen; C: carbon; N: nitrogen; O: oxygen; Si: silicon; Al: aluminum

Figure 1c presents an example of an HN case planned with all three UPRS. The UB (Fig. 1f) is the only one that is always included in the CT simulation. When the proton beam is not designed to pass through the UB, the UB may be excluded from body contour and not calculated in dose calculation. Typically, the UB is placed so that its inferior ends are close to the patient’s shoulders while remaining comfortable. This is to ensure the lateral field can cover sufficient low neck targets during proton planning without approaching the UB corner. Generally, ALB (Fig. 1e) and CTB are manually added to the plan. The Hounsfield units (HU) of UPRS are overridden with proper values that provide the same range pullback to what is measured. In this example, although there is no beam passing CTB, it is also added and included in the dose calculation volume to increase the freedom in choosing proton beam orientation. All three UPRS are placed at the closest position to the patient and maintain minimal air gaps. UPRS and MRS placement can be checked by using in-house software for potential collisions [10].

**Fig. 2:**
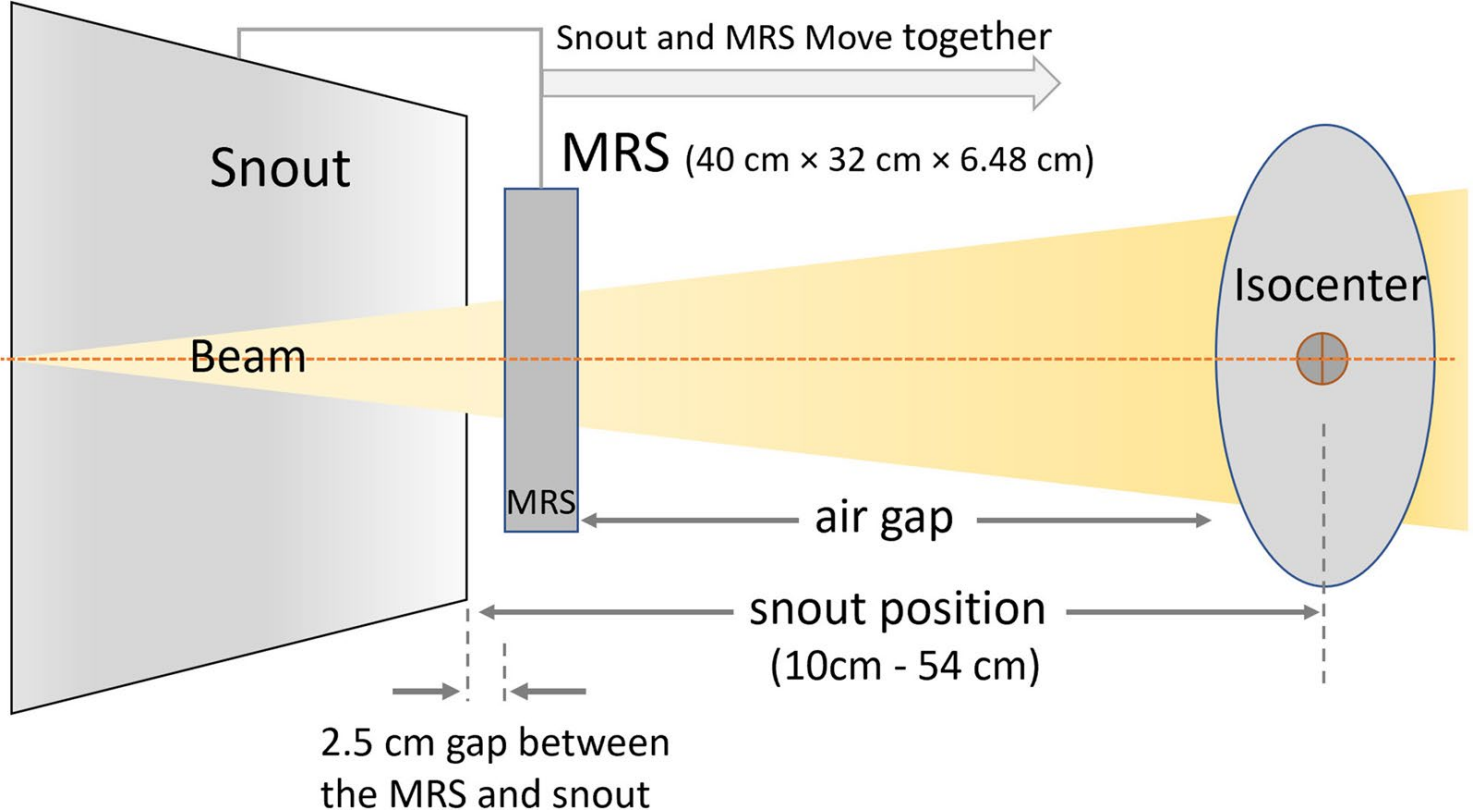
A schematic diagram of a pencil beam scanning proton beam snout with a machine-related range shifter (MRS: 40 cm × 32 cm). The air gap is defined as the distance from the isocenter to the MRS. The snout position is defined from the isocenter to the bottom edge of the snout. A 2.5 cm gap exists between the MRS and the edge of the snout. The snout can be extended towards the isocenter. The range of the snout extensions is from 10 to 54 cm with a fully retracted position at 54 cm

Three sets of data for in-air spot size were measured and compared in this study. The air gap and snout position for our system is described in Fig. 2. Firstly, the spot profiles for open field and with MRS at different air gaps (6.0 cm, 16.0 cm, and 26.0 cm) from the isocenter were measured using a Lynx (IBA-Dosimetry, Schwarzenbruck, Germany). Then a simulation study was carried out using an analytic approximation method (“differential Molière” formula) described by Shen et al., [11] for a few air gaps including the ones described above (air gaps of 2.0 cm, 6.0 cm, 10.0 cm, 16.0 cm, and 26.0 cm). To explore the limitation of TPS, a single proton spot with a nominal energy of 110 MeV and 200 MeV was calculated by the analytic dose calculation algorithm in Eclipse (Pencil Beam Convolution Superposition, version 13.7, Varian Medical Systems) within a phantom for a UPRS with WET of 7.35 cm placed at various locations with air gaps of 6 cm, 16 cm, 26 cm, and 33 cm. For a given energy, the same planning parameters such as the beam geometry, monitor units, and dose calculation volumes were used. Comparisons of lateral profiles at the phantom surface and the percent depth dose curves were generated and analyzed.

To explore the impact of various air gaps on the delivered PBS dose, a single-field optimized PBS field (range 28 cm (~ 210 MeV), modulation 13 cm) was delivered three times on solid water phantom with the ALB placed at 0, 10, and 20 cm air gaps to the phantom surface. The two-dimensional dose maps at the middle of the spread-out Bragg peak were measured using the ion chamber array MatriXX PT (IBA-Dosimetry, Schwarzenbruck, Germany) located at isocenter. Measured dose maps were compared to the dose map calculated from Eclipse. Evaluation of gamma passing rates (criteria of 3%, 3 mm with a threshold of 10%) was also carried out for all three measurements.

The 6.20 cm WET UB (Fig. 1f) was designed specifically for HN and brain targets. It allows lateral to vertex treatment fields with small air gaps and has no clearance issue as shown in Fig. 1c. Generally, the patient is immobilized with a thermoplastic mask and the UB can be inserted in two tracks on the treatment couch top with designed indexes along the cranial-caudal direction. During daily treatment, once patient alignment is completed with orthogonal kV images, the UB is inserted to the indexed locations which were decided during the CT simulation. Due to the irregular shape of the UB, the relative position between the UB and the patient is confirmed with orthogonal kilovolts (kV) images by comparing it with the UB contour in the treatment plan, noting especially the edge of the UB. If an oblique field is used(e.g. the right-posterior oblique field in Fig. 1c) the field edge to the end of the UB should be carefully checked. We recommend at least a 1 cm buffer distance to be maintained from the field edge to the bolus edges during the planning stage.

The 7.35 cm WET CTB permits a posterior or posterior oblique proton field to treat shallow targets. The couch top (QFix, Avondale, PA, USA) is capable of sliding longitudinally. With four indexed sliding positions, the couch top permits an extra 63 cm longitudinal extension. This is extremely important for treating long and posterior shallow target volumes such as craniospinal treatments with PBS technology [12]. To reduce the potential uncertainties, a large oblique angle is prohibited to prevent the beam from passing through the couch corner. If a large oblique beam is required, the distance from isocenter to couch edge must be compared to the distance in the treatment plan, typically provided by the planner through a setup note.

The 7.16 cm WET ALB allows proton beams to be delivered from anterior or lateral directions for shallow targets. ALB includes two rectangular slabs (anterior: 40 cm × 60 cm; lateral: 40 cm × 40 cm) that are attached to a movable cart (Fig. 1a). ALB is the only UPRS that has limited ability to adjust the air gap to the patient surface for anterior beams. Three indexed vertical locations 10 cm apart are available to maintain a small air gap and avoid a collision. The distance between the bottom of the bolus and the surface of the couch top is used for every anterior proton beam to verify the consistency between planned and treatment positions of ALB. To minimize the total weight of ALB itself, only one lateral RS is inserted at any time and it can be manually switched to the opposite side if needed. To reduce their impact on imaging quality, the UB and ALB are removed during the patient initial alignment using orthogonal kV images, while the CTB always remains and the optimized imaging parameters are required to maintain a reasonable anterior–posterior imaging quality. Before the delivery of a field, a therapist reads through all the planned devices and their associated parameters and another therapist confirms all these values.

## Results

**Fig. 3:**
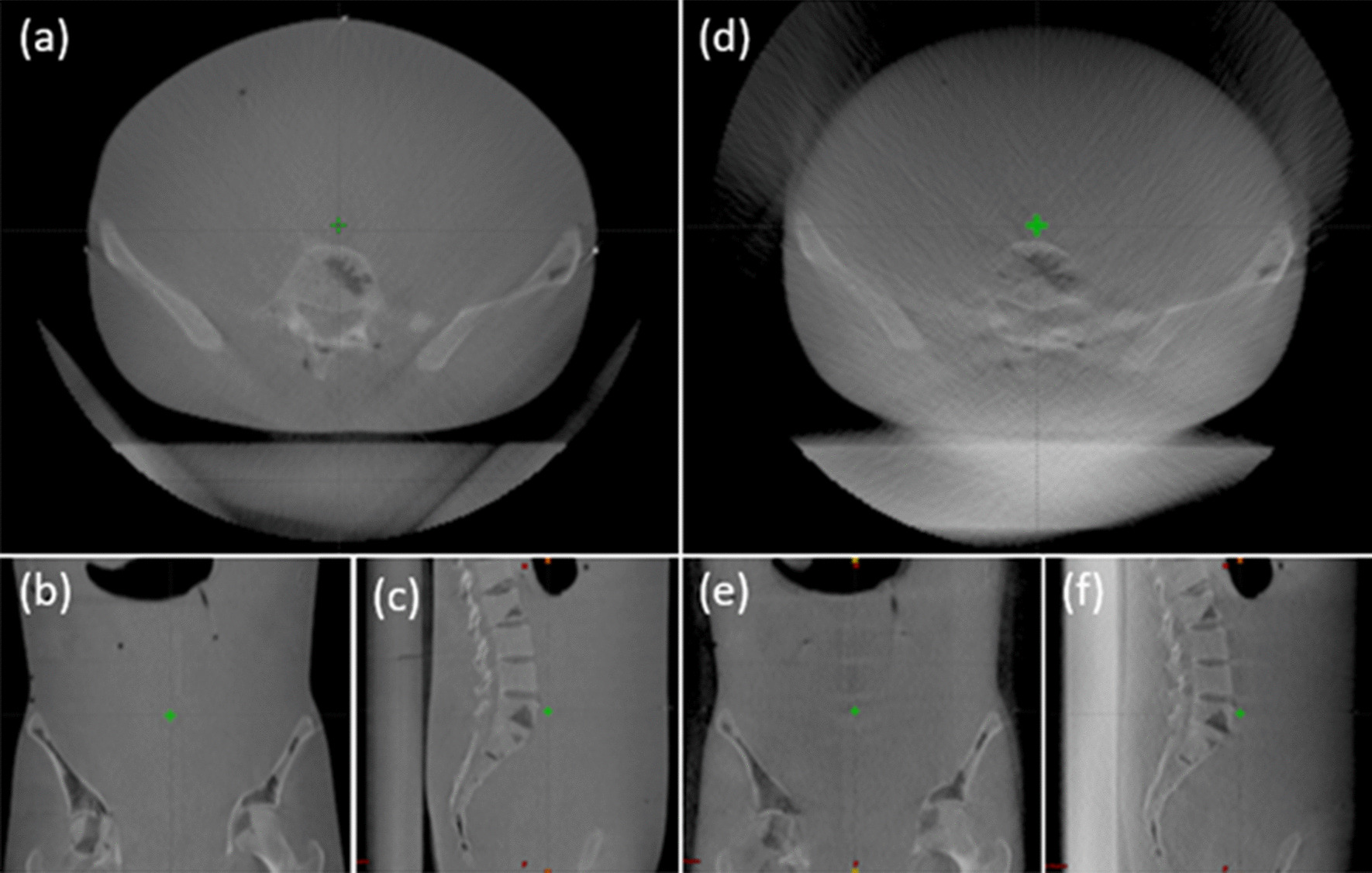
**a**–**c** CBCT images of the RANDO phantom on a 7.5 cm solid water phantom; **d**–**f** CBCT images of the RANDO phantom on a 3.5 cm G-10 CTB (7.35 cm WET). Artifacts are observed and the bony structures are blurred in the axial view. The green crosses in the images represent the imaging isocenters and the similar isocenter locations indicate the same CBCT scanning geometry

Different types of UPRS materials may have a different impact on the verification imaging quality, in particular on volumetric imaging such as cone beam computed tomography (CBCT). As the thick couch top is always present, high Z materials [for example G-10 fiberglass (Table 1)] in the couch can cause artifacts degrading the image quality. This kind of degradation could make it a challenge to identify the implanted fiducial markers or bony structures such as a vertebral body during the patient alignment with kilovoltage orthogonal images. Figure 3 presents the comparison of the CBCT images of a pelvis phantom placed on solid water phantom and CTB with the same WET. Both images were collected using the pelvis scanning protocol and presented in the same window/level settings. Image degradation can be seen especially in the axial view.

**Fig. 4:**
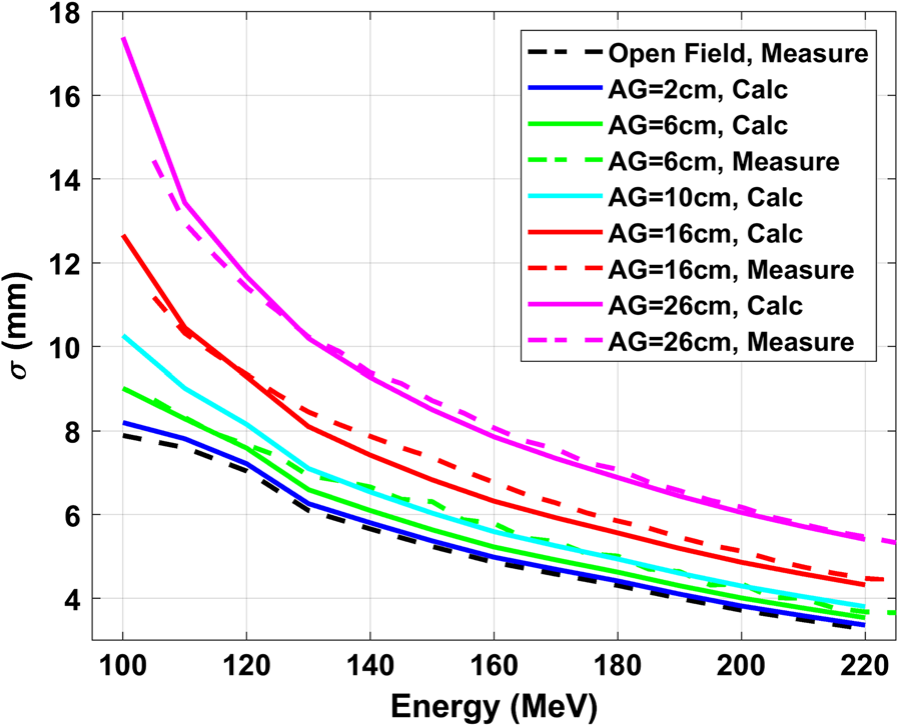
PBS spot sizes versus proton energy from measurements at the isocenter for the open field (red), MRS with air gaps (AG) of 6, 16, and 26 cm (black), and simulation with AG of 2, 6, 10, 16, and 26 cm respectively, which were calculated with the differential Moliere formula. The AG was selected based on typical treatment conditions. UPRS can generally be placed as close to the patient's surface as 2–10 cm AG. To avoid collision between MRS and patient, the AG for brain and head and neck treatment are typically 15–20 cm. The largest AG of 35 cm was selected to represent the worst-case scenario

As shown in Fig. 4, both measurements and Moliere simulation show that the spot sizes increase with larger air gaps and the increases are much more significant for low energy protons. The UPRS, especially UB and CTB, can always be placed close to the patient; therefore, spot sizes don’t change significantly relative to the open beam (less than 2 mm change for air gaps of 2 to 10 cm). MRS would have the same effect for small air gaps. Small air gaps are achievable with MRS with no couch rotations and the snout can be brought close to the patient without causing collisions. However, it may not be always achievable for fields with large oblique angles or associated with complex patient geometries. For example, the small air gap for the lateral field for brain and HN treatment with MRS may cause a collision with the treatment couch or patient shoulder. Therefore, the air gap may increase up to 15 cm (a maximal increase of 4.5 mm on spot size). Often the air gap predicted by the TPS may not reflect the true treatment air gap and collision can still occur between the nozzle and the patient’s body or the immobilization device that is not included in the CT scan. To deliver the planned beam, the snout must be pulled back and the air gap must be increased. As a result, the delivered treatment may be suboptimal.

**Fig. 5:**
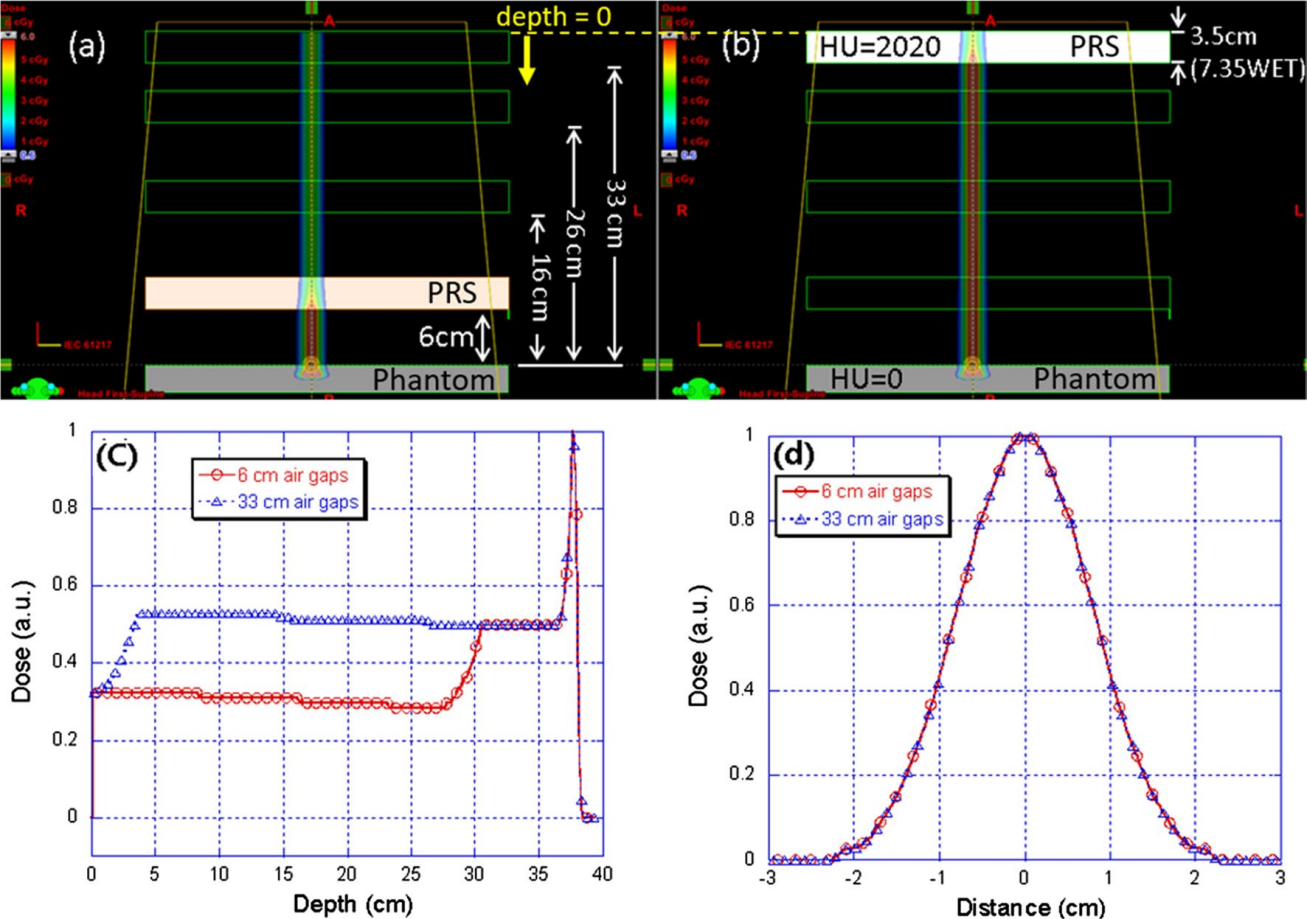
Comparison study for 110 MeV single proton spot with UPRS at different locations (indicated by the green boxes) in Eclipse TPS. **a** Planning geometry with 6 cm air gaps; **b** planning geometry with 33 cm air gaps. **c** the percent depth dose curves for 6 and 33 cm air gaps; the depth 0 cm corresponds to the entrance of the beam, which is the top surface of the first rectangular contour along beam direction (yellow dash line). **d** spot profiles at the phantom surface for 6 and 33 cm air gaps

As shown in Fig. 5, the comparison studies in Eclipse show no change in spot size or percent depth dose (PDD) when the air gaps increase from 6 to 33 cm. The same PDDs are expected as shown in Fig. 5c since the same RS is placed in the beam path and should cause the same amount of pullback on the range of the protons. Thus, the protons should eventually stop at the same depth. However, the trend of an increase in spot size with larger air gaps presented in Fig. 5 is not found in the comparison study in Eclipse. According to Fig. 5d, Eclipse does not consider the effect of the various air gaps on the spot size when a single proton spot travels in between the RS and patient. Here PDDs and spot profiles for air gaps of 6 cm and 33 cm are presented, but results for the other two intermediary simulated air gaps of 16 cm and 26 cm are found to be consistent with that of the two extreme conditions.

**Fig. 6:**
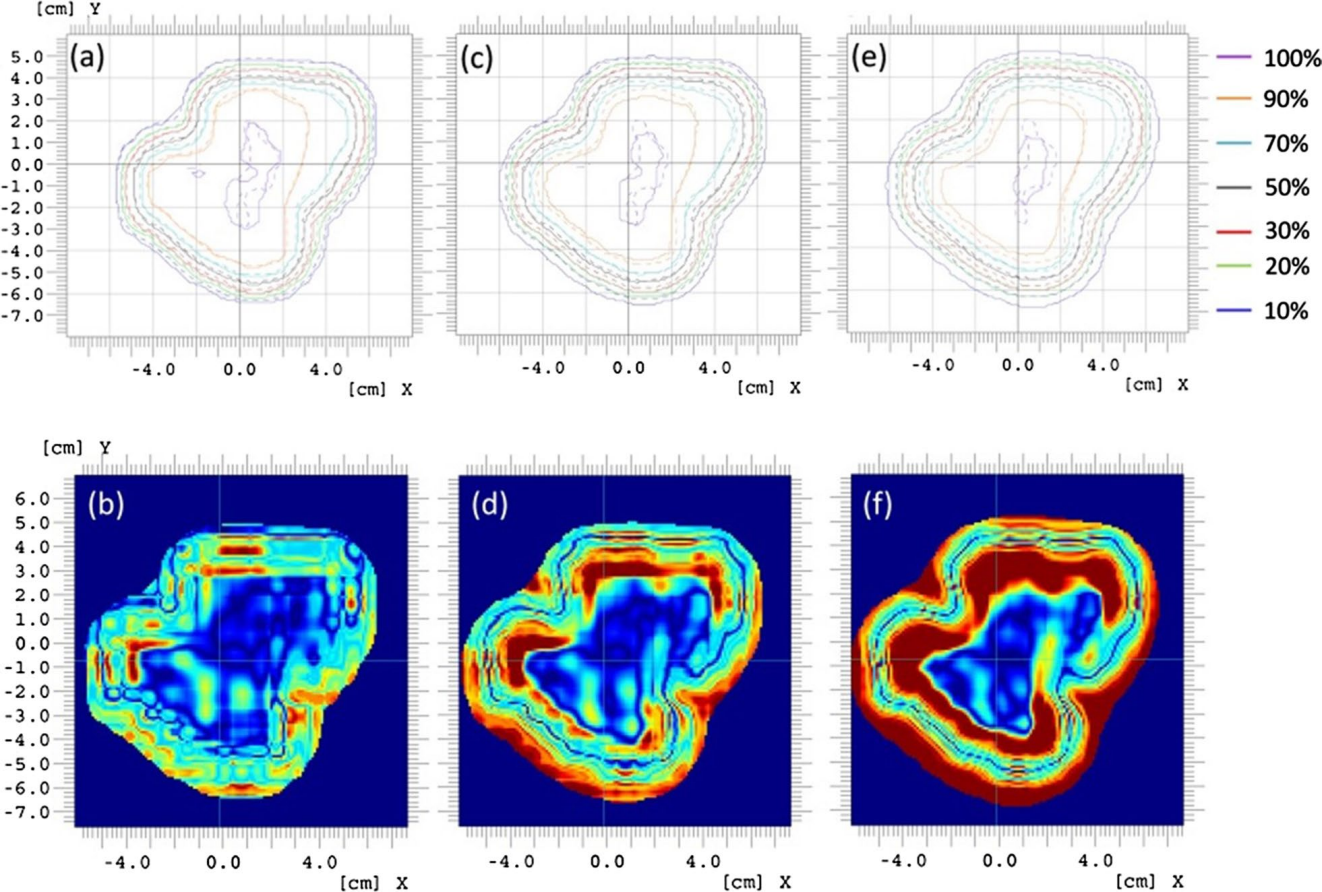
The comparison of iso-dose levels (**a**, **c**, **e**) and gamma analysis (**b**, **d**, **f**) between planed (dash iso-dose lines) and measured (solid iso-dose lines) dose of a PBS field with air gaps of 0 cm (**a** and **b**), 10 cm (**c** and **d**) and 20 cm (**e** and **f**)

Dose deviations between measured and planned dose maps for increased air gaps were found in iso-dose level comparisons and gamma evaluations (3%/3 mm), as shown in Fig. 6. When no air gaps exist between ALB and phantom, a great agreement was observed (Fig. 6a) and the gamma analysis shows 99.5% of the points of interest pass the comparison criteria. As the air gap increases to 10 cm, iso-dose lines start to deviate from the calculated ones. As clearly shown in Fig. 6c, the 90% isodose line shrinks and the 10% isodose line expands, which becomes more significant when the air gap increases to 20 cm (Fig. 6e). The failure rate of gamma evaluation increases from 0.5% to 5.73% and 25.5% when the air gaps increase from 0 to 10 and 20 cm respectively. Most of the failures occur in the dose regions of 80% to 100% and 10% to 30% of the prescribed dose, where the dose gradient changes. As indicated in Fig. 4, the spot size increases with air gaps for all energy levels. Regardless of the size of the air gap, each spot carries the same integral dose, but the dose spreads laterally more with a larger air gap. As a result, the dose changes little at the center of a uniform dose region due to dose equilibrium as dose loss from a single spot is compensated by dose tails of the surrounding spots. The equilibrium is broken at the region where the dose gradient changes (80–100% dose) which can potentially cause under-coverage of the target volume. At the field edge, the dose spreads out further with increased air gaps due to larger spot sizes potentially resulting in a higher dose to surrounding OARs. In this current study, we did not model the air gaps for ALB in the treatment planning system, which contributed to the low passing gamma rate.

## Discussion

A small spot is especially critical in maintaining a high quality of treatment for HN and brain targets where critical organs are often close to the target volume. Quan et al. evaluated the multi-field and single-field optimization for PBS treatment of HN cancer, which reported that with smaller spot sizes, better normal tissue sparing without sacrificing target coverage can be obtained [13]. Wang et al. studied the impact of spot size on plan quality of PBS radiosurgery for peripheral brain lesions and concluded that the spot size at the patient surface must be small to achieve comparable or smaller brain necrosis normal tissue complication probability (NTCP) relative to photon radiosurgery techniques [4]. Kang et al. presented a case using a patient-specific bolus to maintain a small spot size during the treatment of periorbital disease [14]. Van de Water et al. investigated salivary gland sparing in oropharyngeal cancer for PBS technique with a reduced spot size and concluded that reduced spot improved sparing of the salivary glands and reduced NTCP for xerostomia and parotid and submandibular salivary dysfunction [15]. Kralik et al. demonstrated that the smaller spot size, afforded by a PBS-dedicated nozzle relative to a universal nozzle, allows sharper dose distribution fall-off around the target and greater adjacent normal tissue sparing for pediatric brain tumors [16]. Therefore, the use of UB, which consistently reduces the air gap to the patient's surface and maintains the integrity of the original spot, is beneficial for HN and brain proton therapy with the PBS technique.

The movable nozzle with MRS can bring the range shifters closer to the patient. In a clinic, an air gap of 10–20 cm is often employed. It is not rare to see during planning that a large air gap must be used to avoid collision between the bulky gantry and the patient, or the patient immobilization device or the couch. Especially, when the scanned length of a CT image is limited, a plan with a more conservative air gap is preferred to avoid complications on the treatment day. For example, a collision between the gantry and the patient’s arms when the patient’s arms are up above the head, or a breath-hold device can occur for a breast or thoracic patient when the patient’s arm or breath-hold device is not included in the CT scan. Another common example is the collision between the nozzle and the couch at a lateral beam or posterior oblique beam angle for an HN treatment when the planer tries to achieve a small air gap during the planning stage. In contrast, when a UPRS, especially for UB and CTB is used, the air gap can be reduced to a few cm (very challenge for MRS) and there is no concern about the potential collision. When a patient is treated with an oblique beam angle or a couch rotation, the reduction of the air gap will be even larger.

The application of the three-dimensional (3D) printing technique to radiation therapy is of increasing interest and application [17]. 3D printed devices have been used to modulate proton beam energy [18] and served as a customized bolus for passive-scattering proton beams. Because of the high precision of 3D printing, this technique can produce a device that best fits the shape of a patient's body such as the head and extremities. Besides acting as an immobilization device, the device can also serve as RS to allow proton treatment to shallow targets. The use of 3D-printed RS has its unique benefits including negligible air gap and patient or beam specific design. As a non-removable device, 3D-printed RS may cause a challenge in imaging the patient similar to CTB. However, multiple printing materials are available on the market, and the materials such as PLA and polyamide, without containing any high Z components, potentially have little impact on volumetric imaging quality. As described by Zou [19], the 3D printing technique can typically achieve high precision during the manufacture of a device, which is critical for proton therapy. With lower cost and improved printing efficiency, this 3D printing application in PBS radiotherapy is promising.

All the UPRS devices are included in the patient body contour to be accounted for accurately in the proton dose calculation, as shown in Fig. 1c. Small air gaps (< 10 cm) could typically be maintained for UB and CTB, while the determination of the air gaps between the ALB and patient rely on our experience (such as treatment site and patient position) and collision may be predicted by in-house program [10]. Compared to the MRS, the UPRS devices are not interlocked with the beam delivery system. In some cases, multiple UPRS and MRS are used for a single treatment. Thus, effective procedures and clinic workflow along with enhanced communication within the radiation oncology team must be maintained to provide safe and accurate treatment.

The material, dimension, and weight of the UPRS should be considered when the UPRS is designed. Shen reported the impact of the range shifter material on PBS spot characteristics and show that Lexan and Lucite are desirable range shifter materials as their scattering power is similar to water [11]. The minimal energy of a proton system determines the maximal thickness of an RS required to treat a surface target. Thinner RS CTB and UB that are attached to the couch system may reduce the total weight limit of the system. For example, the CTB reduces the weight limit of 440 lbs. for the regular QFix treatment couch to 350 lbs. When a UB (25 lbs.) is employed, the total weight limit is further reduced. To maintain a reasonable weight limit, the ALB is designed to be independent of the couch system. Besides, the use of UPRS often restricts proton beam angles. For example, CTB only allows a posterior field with a limited oblique angle. Due to the flat surface and the limited dimension of the RS, a large oblique angle or couch kick may not be feasible for ALB. In contrast, MRS provides more freedom on beam angle selection while the air gap may depend on an individual case. Some current proton systems allow minimal proton energy of 70 MeV, which corresponds to a WET of 4.1 cm. As a result, the scattering effect, potential collision, reduction of the weight limit of the patient supporting system, and the degradation of image quality from a bulky RS are lower.

HN planning is always one of the challenges in radiotherapy due to the complex target shapes and the proximity of OARs to target volumes. To achieve the best target coverage and maximal sparing of surrounding OARs, the smallest proton beam size has to be maintained for PBS treatment. Thus, air gaps need to be kept as small as possible. To pursue this goal, we standardize the HN planning with two posterior oblique fields and one anterior field with a gradient dose match [20]. For unilateral and simple target volume (for example, in Fig. 1c) optimal field angles (short and simple beam path) other than standard field geometry along with proper RS may be selected based on the size and location of the target.

Recently, Lin et al. [21] evaluated the accuracy of the AcurosPT algorithm, a commercial Monte Carlo algorithm in the Eclipse 13.7 TPS. Although the new algorithm showed a faster increase of spot size along with depth than measurements due to overestimated scattering effect, high consistency between the calculated and measured spot size at the phantom surface was reported for different air gaps. These results indicated that the relationship between the air gap and spot size was accurately modeled by this new algorithm in the next version of Eclipse, which provided improved accuracy on proton dose calculation.

Since our group deployed the first in-room volumetric imaging in proton therapy [22], fixed UPRS devices may not be desirable as the interference to the image and clearance issue may decrease the utility of the device. The image quality of CBCT is limited by scattered radiation [23], reconstruction artifacts from high-density objects [24], and objects extending outside the scanning field of view [25]. The fixed UPRS affects the image quality in various ways as X -rays pass through the RS slab. Firstly, the x-ray beam gets attenuated; secondly, scattered radiation increases; thirdly, UPRS extended outside the field of view leads to reconstruction artifacts. Besides UPRS like CTB, containing high-density materials introduces more artifacts to the CBCT image and further degrade the image quality. However removable UPRS such as the Universal Bolus [3] may provide superior dose distribution while not affecting volumetric imaging, although the workflow would have to be modified.

## Conclusion

We have described how to implement UPRS and MRS for various clinical indications using the PBS technique, and comprehensively reviewed the advantage and disadvantages of UPRS and MRS. We recommend the removable UB only to be employed for the brain and HN treatments while an automated MRS is used for all proton beams that require RS but not convenient or feasible to use UB. During the development of UPRS, its impact on the imaging system should be investigated. While using MRS, it is essential to verify that the TPS accurately models the air gap.

## Data Availability

Not applicable.

## Abbreviations

RS: Range shifter
MRS: Machine-related range shifter
UPRS: Universal patient-related range shifter
UB: U-shaped bolus
ALB: Anterior lateral bolus
CTB: Couch top bolus
IGRT: Image-guided radiation therapy
PBS: Pencil beam scanning
HN: Head and neck
TPS: Treatment planning system
PCS: Proton convolution superposition
WET: Water equivalent thickness
CT: Computed tomography
HU: Hounsfield units
CBCT: Cone-beam computed tomography
PDD: Percent depth dose
NTCP: Normal tissue complication probability
3D: Three dimensional
